# Dynamic Changes in the Thylakoid Proteome of Cyanobacteria during Light-Regulated Thylakoid Membrane Development

**DOI:** 10.3390/plants12233967

**Published:** 2023-11-25

**Authors:** Fang Huang, Arturas Grauslys, Tuomas Huokko, Eva Caamaño Gutiérrez, Andrew R. Jones, Lu-Ning Liu

**Affiliations:** 1 Institute of Systems, Molecular and Integrative Biology, University of Liverpool, Liverpool L69 7BE, UK; agraus@liverpool.ac.uk (A.G.); ttthuo@utu.fi (T.H.); caamano@liverpool.ac.uk (E.C.G.); jonesar@liverpool.ac.uk (A.R.J.); 2 Computational Biology Facility, Liverpool Shared Research Facilities, University of Liverpool, Liverpool L69 7ZB, UK; 3 College of Marine Life Sciences, and Frontiers Science Center for Deep Ocean Multispheres and Earth System, Ocean University of China, Qingdao 266003, China

**Keywords:** cyanobacteria, photosynthesis, acclimation, thylakoid membrane, membrane biogenesis, proteome

## Abstract

Cyanobacteria were among the oldest organisms to undertake oxygenic photosynthesis and have an essential impact on the atmosphere and carbon/nitrogen cycles on the planet. The thylakoid membrane of cyanobacteria represents an intricate compartment that houses a variety of multi-component (pigment–)protein complexes, assembly factors, and regulators, as well as transporters involved in photosynthetic light reactions, and respiratory electron transport. How these protein components are incorporated into membranes during thylakoid formation and how individual complexes are regulated to construct the functional machinery remains elusive. Here, we carried out an in-depth statistical analysis of the thylakoid proteome data obtained during light-induced thylakoid membrane biogenesis in the model cyanobacterium *Synechococcus* elongatus PCC 7942. A total of 1581 proteins were experimentally quantified, among which 457 proteins demonstrated statistically significant variations in abundance at distinct thylakoid biogenesis stages. Gene Ontology and KEGG enrichment analysis revealed that predominantly photosystems, light-harvesting antennae, ABC transporters, and pathway enzymes involved in oxidative stress responses and protein folding exhibited notable alternations in abundance between high light and growth light. Moreover, through cluster analysis the 1581 proteins were categorized into six distinct clusters that have significantly different trajectories of the change in their abundance during thylakoid development. Our study provides insights into the physiological regulation for the membrane integration of protein components and functionally linked complexes during the cyanobacterial TM biogenesis process. The findings and analytical methodologies developed in this study may be valuable for studying the global responses of TM biogenesis and photosynthetic acclimation in plants and algae.

## 1. Introduction

Photosynthesis is an essential biological process on the planet. Through photosynthesis, solar energy is captured by photoautotrophic organisms, including plants, algae, and cyanobacteria, and converted to chemical energy which can be directly used by living cells. Cyanobacteria are the oldest oxygenic photoautotrophs and are widely distributed on the planet, serving as a predominant contributor to the global carbon cycle and primary production [1,2,3]. The cyanobacterial thylakoid membrane (TM) is a sophisticated system that accommodates protein complexes involved in both photosynthetic and respiratory electron transfer chains, resulting in the physiological operation and regulation of oxygenic photosynthesis and respiration within the same intracellular membrane [4,5]. The photosynthetic electron transport complexes in cyanobacterial TMs involve the phycobilisome (membrane-associated light-harvesting antenna supercomplex), photosystem II (PSII), photosystem I (PSI), cytochrome (Cyt) *b*_6_*f*, and ATP synthase (ATPase) [4,6]. The main respiratory electron transport complexes include type-I NADH dehydrogenase (NDH-1), type-II NAD(P)H dehydrogenase (NDH-2), succinate dehydrogenase, cytochrome oxidase, and alternative oxidases. There are small electron transport molecules, such as plastoquinone, plastocyanin, and cytochrome *c*_6_, which act as electron carriers to shuttle electrons between each electron transport complex and functionally link all the complexes together [7]. These molecules, together with Cyt *b*_6_*f*, are shared between photosynthetic and respiratory electron transport pathways [8]. Moreover, cyanobacterial TMs contain assembly factors, ion channels, and receptors, which are responsible for membrane complex assembly, TM biogenesis, and functional regulation.

Light is not only a key motive force driving photosynthetic electron transport but also an indispensable regulator of photosynthetic and metabolic activities occurring in photoautotrophs. The environmental changes in light intensity and wavelength have been shown to make a significant impact, at either short-term (seconds to minutes) or long-term (days to weeks) timescales [9], on the light-absorption capacity and photosynthetic rate, chlorophyll biosynthesis, TM composition and architecture, expression and assembly of photosynthetic complexes, TM lateral protein arrangement, state transitions, photoprotection, as well as phototaxis [10,11,12,13,14,15,16,17,18,19,20,21]. Despite the advanced knowledge, how cyanobacterial cells regulate the abundance and assembly of their TM and associated proteins during light adaptation and during the thylakoid membrane biogenesis process remains less understood.

**Figure 1:**
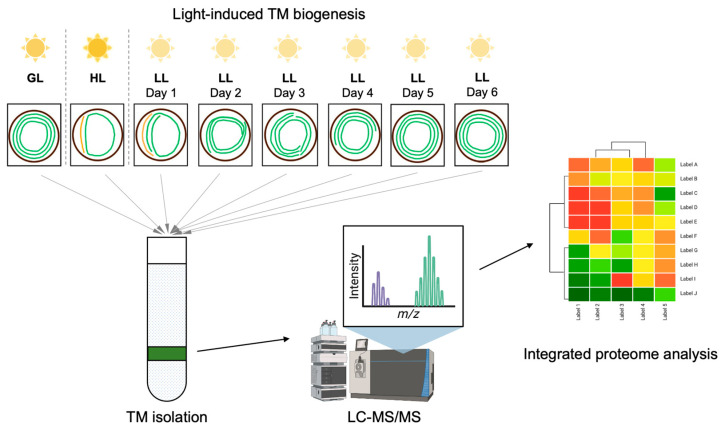
The overall workflow of light-regulated Syn7942 thylakoid biogenesis and integrative proteome analysis. Syn7942 cells were cultured under continuous growth light (GL) for 5 days and treated with high light (HL) for 14 days until the stable minimal level of TM was reached. From that time point, TM regeneration was initiated by shifting cells to low light (LL) for 6 days until full recovery, and sample cells were harvested on each day (LL1–LL6). The TM content (green curved lines) per Syn7942 cell (circiles) varies depending on the intensity of light illumination. Those samples were used for TM fractioning procedure and the following mass spectrometry detection as well as proteome analysis.

The regulation of light intensity, in combination with proteomic characterization, has provided powerful tools for studying TM biogenesis and development processes in great detail [9,10,22]. Previous studies grew dark-adapted *Synechocystis* sp. PCC 6803 (Syn6803) cells under light-activated heterotrophic growth conditions to promote TM regeneration [22]. Analysis of the proteome of Syn6803 allowed quantitative identification of 641 proteins, among which the proteins exhibiting significant changes in abundance were associated with oxidative stress and heterotrophic growth-related metabolism and carbon/nitrogen balance [22]. However, TM fractions are only a small part (24.5%) of the total proteins identified, making it challenging to perform a comprehensive analysis of the TM biogenesis process and dynamics. Our recent work developed a method to modulate TM biogenesis in *Synechococcus elongatus* PCC 7942 (Syn7942) by adjusting light intensities during cell growth (Figure 1) [10]. The high light (HL)-acclimated Syn7942 cells exhibited minimal TM content, and the following switching to low light (LL) promoted the regeneration of TM. We performed proteomic characterization of the TM fractions isolated from Syn7942 cells that grew under different light conditions, using liquid chromatography–tandem mass spectrometry (LC-MS/MS) combined with label-free quantification. This approach allowed us to determine the changes in protein content of major photosynthetic complexes during Syn7942 thylakoid biogenesis [10].

In this study, we conducted an in-depth statistical analysis of the above obtained global Syn7942 thylakoid proteome data and studied the dynamic changes in protein abundance of TM (and associated) complexes during HL and LL adaptation. Our results provide insights into the physiological modulation of the biosynthesis and membrane integration of protein components during TM biogenesis.

## 2. Results and Discussion

### 2.1. Proteome Data Generation and Processing

The methods used for light-regulated Syn7942 TM biogenesis, TM isolation, and proteomic measurements were described previously [10]. The proteome data of TMs isolated from cells grown under different light conditions (growth light, GL, 40 μmol photons m^−2^ s^−1^; high light, HL, 300 μmol photons m^−2^ s^−1^; low light, LL, 20 μmol photons m^−2^ s^−1^) are available at the ProteomeXchange Consortium via PRIDE partner repository with the project accession PXD019731 (https://www.ebi.ac.uk/pride/archive/projects/PXD019731 (accessed on 17 November 2020). As a result, we identified a total of 1518 proteins with appropriate quantitative values for statistical comparison (Supplementary Table S1). Our identified proteins represent about 60% of the total predicted proteins in the Syn7942 genome, according to the UniProt database (Proteome ID: UP000889800, 2657 in total) and the Kyoto Encyclopedia of Genes and Genomes (KEGG) genome (T00300, 2661 in total). It is worth noting that the crude membrane fractioning method used in our analysis cannot completely separate TMs from plasma membranes (PMs) and outer membranes (OMs). This was evident by identification of some PM- and OM-integral proteins, for example, the PM-specific NrtA, a component of a nitrate transporter, and OM-specific porins. Nevertheless, our gentle and simple purification strategy appears to be sufficient to maintain the structural integrity of TMs, allowing a global analysis of dynamic events occurring during TM biogenesis. 

The exported data were normalized via probabilistic quotient normalization (PQN) [23]. Data normalization was evaluated by analyzing the boxplots of the samples (Supplementary Figure S1). Before normalization, sample technical variance was noticeable with a batch effect visible on the replicates in the LL-adapted samples (Supplementary Figure S1A). A similar observation was identified by principal component analysis (PCA) (Supplementary Figure S2A). After normalization, the boxplots of all samples showed comparable means and quantiles (Supplementary Figure S1B), suggesting well-normalized data. Although PCA scoring showed a reduced variance in PC1 (Supplementary Figure S2B), there was still noticeable variance between biological replicates. To remove variance associated with technical variance, a batch effect correction was applied and results assessed via PCA showed reduced technical variance and a much clearer structure based on time and treatment (Supplementary Figure S2). These results indicated that high-quality data were obtained for onward analysis.

### 2.2. Statistical Analysis, Pathway and GO Term Enrichment Analysis

Out of the 1518 proteins, 615 could be mapped to the KEGG database and clustered into 16 functional categories, including Carbohydrate metabolism, Energy metabolism, Amino acid metabolism, Glycan biosynthesis and metabolism, Metabolism of cofactors and vitamins, Translation, Replication and repair, Membrane transport, as well as Signal transduction. We evaluated the correlation of all proteins with respect to time, and significant genes were employed for overrepresentation pathway enrichment using the KEGG and Gene Ontology (GO) databases. The KEGG pathway enrichment analysis showed that three pathways were differentially regulated, which are “Photosynthesis-antenna proteins” (KEGG syp00196), “Photosynthesis” (KEGG syp00195), and “ABC transporters” (KEGG syf02010) (Supplementary Figure S3). GO term enrichment analysis showed that the biological process “Photosynthesis” and cellular component “Thylakoid membrane” were significantly enriched, suggesting that these processes and components are active during TM biogenesis.

### 2.3. Characterization of Identified Proteins

**Figure 2:**
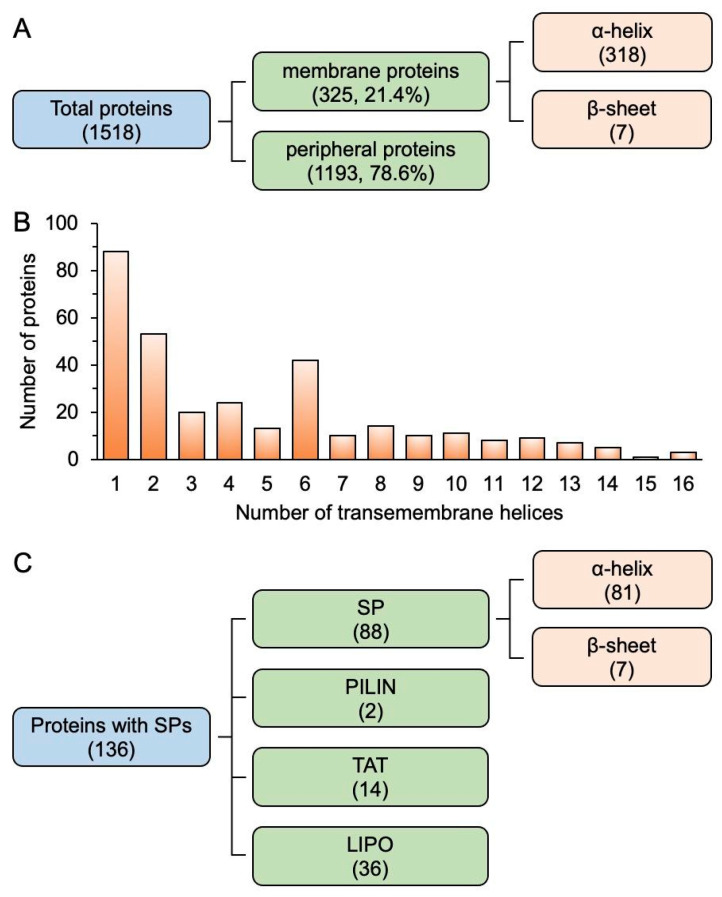
Topological characterization of membrane proteins from Syn7942. (**A**), Quantification of proteins based on types predicted by using DeepTMHMM. (**B**), Frequency of the number of transmembrane α-helices in proteins. (**C**), Quantification of proteins with different signal peptides predicted by SignalP.

To characterize the membrane proteins by structure, protein topology was predicted by DeepTMHMM [24] and the presence of signal peptides (SPs) was predicted by SignalP. Important characteristics, including protein types (integral or peripheral), number of transmembrane helices (TMH), SP, and protein orientation, were analyzed on 1518 proteins (raw data in Supplementary Table S2). The analysis showed that 325 proteins (21.4% of all mapped proteins) were identified as integral membrane proteins, among which 318 proteins have α-helical transmembrane domains and 7 have β-sheet transmembrane domains (Figure 2A). Within the 318 transmembrane proteins, the top three α-helices numbers most frequently present in proteins are one, two, and six, together forming 58% of all helical transmembrane proteins (Figure 2B). Four of the seven uncharacterized proteins with β-sheet transmembrane domains are presumably porin proteins, which are typical beta barrel outer membrane proteins that allow passive diffusion of metabolites across membranes.

Among the 1518 proteins, 136 proteins contain SPs predicted by SignalP (Supplementary Table S3). Figure 2C shows that 88 proteins are predicted to have N-terminal SP sequences that target proteins to the secretory (Sec) pathway for translocation across the PM in prokaryotes [25], 14 proteins have twin-arginine translocation (TAT) signal, 36 are lipoproteins, and 2 have pilin SPs. These results indicate that the majority (65%) of proteins with SPs are directed to their specific cellular locations via the Sec pathway, including those that are released into the extracellular space or incorporated into cell membranes.

### 2.4. The Proteome of HL-Adapted Cells

**Figure 3:**
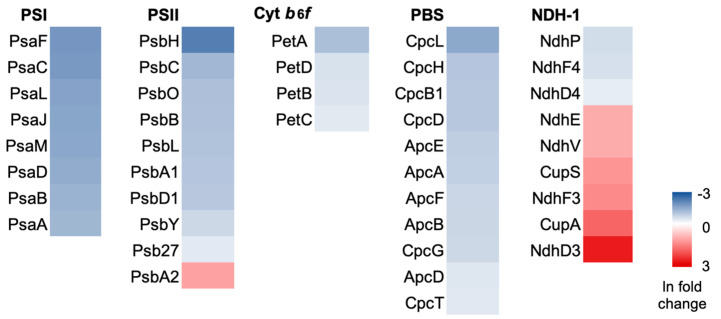
Heatmap of photosynthetic protein expression pattern under HL adaptation.

To study the HL acclimation, Syn7942 cells were treated with HL for 14 days until the stable minimal level of TM was reached and TMs were then isolated from the HL-adapted cells for mass spectrometry analysis [10]. Univariate statistical analysis of the proteome data showed that the content of 457 out of 1518 proteins was statistically significant (adjusted *p*-value < 0.05). Among these proteins, 439 proteins were differentially regulated under HL compared with GL, using a cutoff of 1.5-fold change (log2FC between −0.405 and 0.405) (Supplementary Table S4), suggesting that these 439 proteins are more responsive to HL acclimation. Among the 439 proteins, 229 were down-regulated and 210 were up-regulated. In the down-regulated proteins, 58 displayed more than 4-fold abundance reduction (HL/GL ratio < 0.25), including mainly photosynthetic protein subunits and antenna subunits. In the up-regulated proteins, 28 proteins displayed more than 4-fold abundance increase (HL/GL ratio > 4), including transporters, regulatory proteins, and HL-inducible proteins (Supplementary Table S4). A heatmap (Figure 3) illustrates the expression patterns of subunits involved in functionally significant groups listed in Table 1.

#### 2.4.1. Photosystems and Assembly Factors

The protein abundance of three functional groups involved in photosynthetic electron transport, including PSI, PSII, and Cyt *b*_6_*f*, were overall down-regulated under HL (Table 1), except the PSII subunit PsbA2, which functions under HL to replace photodamaged PsbA1 [26,27]. The PSII antenna-associating protein PsbH [28] showed the greatest reduction (HL:GL = 0.09), and other essential PSII proteins were reduced in abundance, with fold change ratios ranging between 0.21 and 0.34. The PSI subunits, including PsaA-D core proteins, PsaF and PsaJ involved in the organization of the PSI complex [29], as well as PsaI, PsaL, and PsaM involved in PSI oligomerization [30], were also reduced in abundance, with the ratios of 0.13–0.21.

The coordinated assembly of polypeptides, integration of various cofactors, and incorporation of light-harvesting antennae are essential for photosystem biogenesis [31]. Assembly factors are required for the stepwise de novo assembly and spatiotemporal organization of a functional PSII [32]. The assembly factors of PSII (including CtpA, Psb27, Psb28, and Psb34) and PSI (including Ycf37 and VIPP1, referred to as IM30) were also down-regulated under HL (Table 1). Other factors involved in PSI stability, including Alb3 (UniProt ID: Q31MS2), Ycf4 (Q31QI3), RubA (Q8KPP5), Hcf101 (Q31P84), and BtpA (Q31K76), were identified in our analysis, but did not pass the thresholds for quantification.

Interestingly, the extent of the reduction of assembly factors was less than that of functional subunits (Table 1). This could be a strategy for Syn7942 to maintain the competence to quickly assemble active photosystem components when encountering rapidly changing growth conditions. Meanwhile, the greater reduction in the abundance of PSI subunits compared to that of PSII subunits would lead to a decreased PSI/PSII ratio under HL acclimation, in agreement with previous observations [33]. The regulation of photosystem stoichiometry (PSI/PSII ratio) allows for optimal photosynthesis under conditions that favor one of the two photosystems, representing an important mechanism for cyanobacteria to tackle environmental stress [34,35,36].

PSII is prone to light-induced damage or photoinhibition under HL [37]. To maintain PSII homeostasis, a repair cycle operates to remove damaged D1 protein (PsbA1) and replace it with newly synthesized D1 protein (PsbA2) [38]. In Syn6803, the photodamaged D1 is removed by the TM ATP-dependent zinc metalloprotease FtsH, which is a hetero-oligomeric complex composed of FtsH2 and FtsH3 [39]. In our analysis, FtsH isomer expression was not induced in response to HL. Instead, other proteases, including ClpP2 (O34125), were up-regulated under HL (Supplementary Table S4), consistent with previous results [40]. Clp proteases are ATP-dependent serine-type endopeptidases. The ClpP1 in cyanobacteria is known to be induced under HL exposure for stress acclimation [41], but the function of ClpP2 in Syn7942 remains still unclear. We propose that ClpP2 might play a role in the degradation of unstable or misfolded proteins induced by HL as a protein quality control system in Syn7942.

#### 2.4.2. Light-Harvesting Antenna

Light-harvesting antennae are essential to efficiently collect solar energy. In cyanobacteria, PBSs are giant and elaborate pigment–protein complexes that serve as major antennae for chlorophyll (Chl)-containing photosystems. Chl biosynthesis enzymes, including light-dependent protochlorophyllide reductase Por (Q935X4), heme oxygenase Hox1 (Q9Z3G6), and protoporphyrinogen oxidase HemJ (Q31PY9), were found to be significantly down-regulated under HL, consistent with previous studies [42]. PBSs are supramolecular complexes comprising phycobiliproteins (PBPs), including allophycocyanin (Apc) in the core and c-phycocyanin (Cpc) rod proteins radiating from the core, as well as anchoring proteins connecting PBSs with PSII to mediate directional energy transfer [43]. The PBSs are attached to hundreds of open-chain tetrapyrrole chromophores (phycobilins) through covalent interaction, a modification catalyzed by lyases [44,45]. PBP subunits, including Apc and Cpc, as well as phycobilin attachment chromophore lyase CpcT [46], were down-regulated under HL (Figure 3, Table 1). Declining antenna content would be expected to lower the susceptibility of the cells to HL damage [42].

These results indicated a concomitant reduction in the abundance of photosystem subunits (Psa and Psb), PBS subunits (Apc and Cpc), and enzymes for pigment biosynthesis (Por and Hem) when adapted to HL. This suggests a close correlation between antenna biosynthesis and the production of their associated photosystem components, enabling cyanobacteria to prevent overabsorption of excessive light energy.

#### 2.4.3. NDH Complexes

Cyanobacterial NDH-1 is the TM-integrated complex involved in CET, respiration, and CO_2_ acquisition [12,47,48,49,50]. NDH-1 exhibits four forms termed NDH-1_1–4_, all of which share a common NDH-1M complex core [47]. Our results demonstrated that protein subunits in different forms of NDH complexes were differently regulated (Table 1). NdhE, the NDH-1M core subunit, was up-regulated under HL with a fold change (FC) of 1.97, suggesting a general increase in NDH-1 content induced by HL. The up-regulation under HL was also found for the subunits specific to NDH-1_3_ that is involved in CO_2_ uptake, including NdhD3 (FC = 12.81), NdhF3 (FC = 3.06), and CupA (FC = 5.06). In contrast, the NDH-1_4_-specific subunits NdhD4 (FC = 0.47) and NdhF4 (FC = 0.38), as well as the NDH-1_1_- and NDH-1_2_-specific subunit NdhP (FC = 0.37), were down-regulated (Table 1). These results suggest that NDH-1_3_ is the main functional form of NDH-1 under HL. Interestingly, the activity of NDH-1 for cyclic electron transport (CET) and CO_2_ uptake under HL is regulated by NdhV [51]. We found an increase in NdhV content under HL (FC = 2.0), in good agreement with the up-regulation of NDH-1_3_, as NdhV is only present in NDH-1_3_ and NDH-1_4_ but not NDH-1_1_ and NDH-1_2_ [51]. As NDH-1 complexes also participate in cyanobacterial CET around PSI [52], NDH-1_3_ could have a preferential involvement in CET during HL adaptation [48]. Both NDH-1_3_ and NDH-1_4_ are responsible for CO_2_ acquisition, and NDH-1_3_ is an inducible, high-affinity uptake system whereas NDH-1_4_ is a constitutive low-affinity uptake system [47]. Our data showed that NDH-1_3_ proteins were up-regulated and NDH-1_4_ components were down-regulated under HL adaptation, consistent with previous proteomic studies [42]. The up-regulation of NDH-1_3_ may suggest the physiological significance of NDH-1_3_ in both electron flow and CO_2_ uptake under HL.

Moreover, the single-subunit NDH-2, which was suggested to play a regulatory role in intersystem electron flow in response to redox state changes in the plastoquinone pool to reduce HL-induced photodamage [47,53], was also significantly induced under HL.

#### 2.4.4. Two-Component Systems

We also identified significant amounts of proteins in two-component regulatory systems, which regulate gene expression and cell behavior to adapt to environmental changes [54]. The Che signal transduction pathway components histidine kinase CheA, response regulator CheY, and CheY-like, as well as CheW, were significantly up-regulated as a response to HL acclimation. On the contrary, the paired SasA/RpaA two-component system, which is involved in the circadian clock-dependent transcriptional regulation [55], was down-regulated under HL.

Proteins involved in the Calvin–Benson–Bassham (CBB) cycle, such as Rubisco subunits and carboxysome components, were not included in this analysis as they are not in the membrane fraction. However, proteins responsible for carboxysome positioning in Syn7942, a two-component system of McdA and McdB [56], were identified to be up-regulated under HL. This is consistent with previous proteomic data showing that components of carboxysomes were induced under HL [42].

#### 2.4.5. Membrane Transporters

Membrane transporters such as ATPases were found both up- (10) and down-regulated (8) (Supplementary Figure S4). The down-regulated ATPases include a copper-transporting P-type ATPase PacS (P37279) and nitrate transporter NrtA (P38043). The former is involved in the maintenance of copper homeostasis [57], which is regulated by the redox status of cyanobacterial cells [58]. The up-regulated ATPases include a ferric iron uptake ABC transporter FutC (Q31ND3) [59] and a high-affinity iron transporter Ftr1 (Q31KG8), suggesting an increased demand for Fe during HL acclimation.

#### 2.4.6. Stress-Related Proteins

HL could induce the production of reactive oxygen species (ROS) that can lead to oxidative damage to cells [60]. The synthesis of antioxidant enzymes is a protective mechanism against ROS [61,62,63]. Some of the enzymes, including membrane-integral thioredoxin (Q31LF0) and glutathione peroxidases (P12608), were significantly up-regulated; while others, including catalase peroxidase KatG (Q31MN3), were down-regulated under HL. These results are in agreement with previous studies in *Synechococcus* sp. PCC 7002 (Syn7002) [42], suggesting that glutathione peroxidase may have an important function in resistance to ROS induced by HL stress through hydrogen peroxide detoxification.

Cyanobacteria also respond to HL stress by accumulating high-light-inducible proteins (Hlips), which are involved in chlorophyll biosynthesis/metabolism and photoprotection in cyanobacteria, algae, and plants [64]. In our analysis, HliA (Q55019) was significantly up-regulated by HL, nearly 10-fold higher than the GL-grown sample, suggesting its importance in mitigating photodamage to Syn7942 cells when there is excessive excitation energy.

#### 2.4.7. Genetic Information Processing

Proteins involved in gene transcription, such as transcriptional regulators (Q31KN3; IdiB, Q31L65) as well as sigma factors SigG (Q31LW6) and SigI (Q31LN5) that confer promoter selectivity, were up-regulated under HL (Supplementary Table S4). The up-regulated proteins also include enzymes involved in DNA repair and replication, such as DNA polymerases (Q31PS7, Q31RU3) and DNA topoisomerase (Q31MJ5), suggesting a high demand for HL-induced DNA damage repair and protein synthesis. In contrast, some other regulators involved in gene transcription (Q31PD3, Q31S27, Q31S42, Q31QE2) were reduced under HL. These transcriptional regulators might regulate gene expression of proteins participating in mechanisms that are minimized such as photosynthesis. Meanwhile, some ribosomal proteins involved in translation were also reduced, suggesting reduced protein synthesis under HL.

### 2.5. Proteome Dynamics during LL Treatment and TM Biogenesis

**Figure 4:**
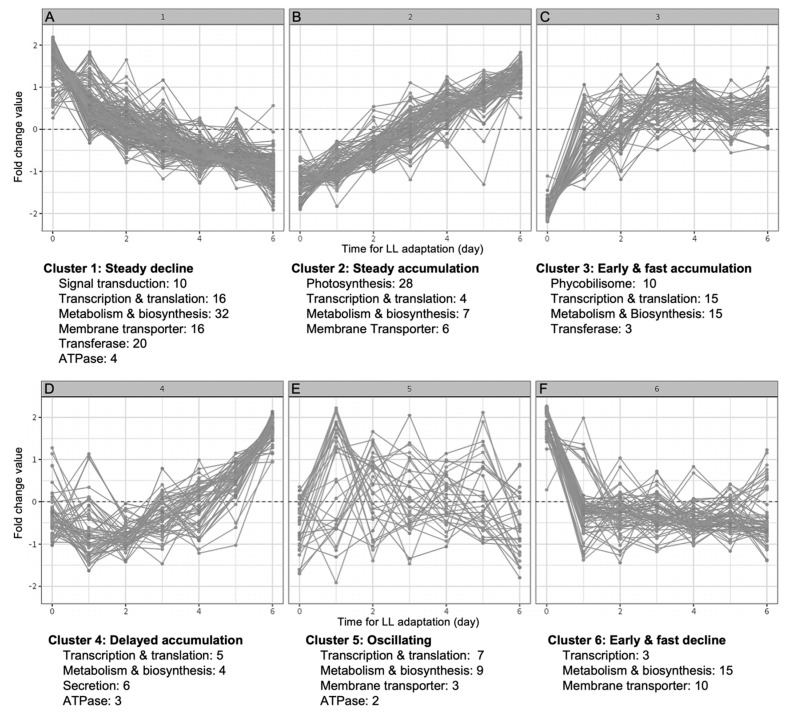
Protein cluster analysis. (**A**), Proteins in Cluster 1 exhibit a gradual decline in abundance during the time course of LL treatment. (**B**), Proteins in Cluster 2 exhibit a gradual increase in abundance during the time course of LL treatment. (**C**), Proteins in Cluster 3 show a fast accumulation at the early stage followed by a stabilization phase around baseline level in the later stage of the LL treatment. (**D**), Proteins in Cluster 4 show a delayed increase at the later stage of LL treatment. (**E**), Proteins in Cluster 5 exhibit no clear trajectories in protein abundance during LL treatment. (**F**), Proteins in Cluster 6 exhibit a fast decline at the early stage and then stabilized around the baseline level at the later stage of LL treatment. Dotted lines indicate zero fold change.

To characterize the dynamic rearrangement of the proteome during LL treatment-induced TM biogenesis in depth, HL-adapted Syn7942 cells were shifted to LL for a continuous growth of 6 days until full recovery of TMs, and sample cells were then harvested on each day (LL1–LL6) for mass spectrometry analysis [10]. Based on the proteome data, we conducted cluster analysis over the 457 proteins that exhibit significant differences between GL and HL (adjusted *p*-value ≤ 0.05). This resulted in the unbiased classification of proteins into six defined clusters according to their trends in abundance changes (Figure 4, Supplementary Tables S5–S10). In each cluster, protein functions were manually assigned different colors based on annotation.

#### 2.5.1. Cluster 1

Cluster 1 was the largest group with 150 proteins (about 1/3 of the total proteins analyzed) (Supplementary Table S5). These proteins exhibited a gradual decline in abundance during the time course of LL treatment (Figure 4). The Cluster 1 proteins are predominantly involved in energy metabolism, including subunits of cytochrome oxidases in the TM-integrated respiratory electron transport chain (Supplementary Figure S5), the NDH-1M subunit NdhE, the inducible NDH-1_3_ subunits NdhD3, NdhF3, and CupA, as well as NDH-2. The general decrease in the contents of NDH complexes suggested reduced respiration activities during LL adaption [10].

In Syn6803, several TM sheets are connected and converge to form contact sites with the PM called thylapses, at which PSII assembly occurs [65]. The attachment of TM to the PM requires the protein anchor of convergence membranes (AncM). Interestingly, the AncM homolog in Syn7942 (Q31MA1) was identified in Cluster 1, but its function remains uncharacterized.

Peptidases are responsible for the processing of SP-containing proteins. Syn6803 peptidase (Slr1377) is involved in the assembly of PSI and Cyt *b*_6_*f* [66]. Its Syn7942 homolog (Q31R00) exhibited a gradually declined content during LL-induced TM biogenesis (Supplementary Table S5).

#### 2.5.2. Cluster 2

Proteins in Cluster 2 exhibited an increase in content after switching from HL to LL (Figure 4). The predominant functional group of proteins in Cluster 2 (28/94) comprises subunits of photosynthetic complexes, including PSI, PSII, Cyt *b*_6_*f*, as well as PSII assembly factors (Supplementary Table S6). According to evidence from Syn6803, at the early stage of PSII assembly, the precursor of the D1 protein is inserted into the PM by the combined action of the insertase YidC and the translocase SecYEG [32]. The periplasmic protein PratA interacts with the C-terminus of D1 and transports Mn^2+^ to D1 [67,68]. Interestingly, these early-stage assembly factors, including YidC and PratA, were not identified in our analysis. This is probably due to their transient feature or limited abundance, or the functional distinction between the two different cyanobacterial species. At the later stage, the C-terminal extension of D1 is cleaved by the processing protease CtpA [69], whose homolog in Syn7942 (Q31KQ9) was identified in Cluster 2. Other known assembly factors for the later-stage PSII assembly, including Psb27 (Q31RE4) and Psb28 (Q31ML0) [70,71,72], as well as the Psb34 subunit (Q31QR0) that is involved in RC47 transition [73], were also identified in Cluster 2. Mn_4_CaO_5_ is an indispensable catalytic cluster of the water-oxidizing complex in PSII. A Mn^2+^ transporter (Q31NM3) was found in Cluster 2, consistent with an increased demand for Mn^2+^. These results reflected a concerted recovery of photosystems, cofactors, and PSII assembly factors, suggesting the importance of coordinated regulation during TM biogenesis.

In contrast, the assembly factors for PSI, including Ycf37 (Q31NR5) and VIPP1 (Q31Q43), were not in Cluster 2 (Supplementary Table S6). Although all showed an increasing trend in response to LL treatment, Ycf37 exhibited a quick response belonging to Cluster 3 (Supplementary Table S7) and VIPP1’s response was delayed belonging to Cluster 4 (Supplementary Table S8). This difference in trend changes may reflect the involvement of different proteins at distinct stages during PSI assembly. Ycf37 is required for the formation of the PSI trimer [74]. The function of VIPP1 varies among cyanobacterial species. It is essential for TM biogenesis in Syn6803 and Syn7942 [75,76,77], whereas its function in Syn7002 was suggested to be mainly involved in PSI biogenesis rather than TM biogenesis [78]. The dynamic changes shown in our analysis suggested that VIPP1 might function at the later stage of PSI biogenesis in Syn7942 (Figure 4D, Supplementary Table S8), compared to the findings from Syn6803 [79].

Cluster 2 also contains the NDH-1_1_- and NDH-1_2_-specific subunit NdhP for NDH-1 complex stabilization [80,81] as well as the subunits of the constitutive CO_2_-uptake system NDH-1_4_ (including NdhD4 and NdhF4). No subunits of the inducible NDH-1_3_ were identified in Cluster 2.

#### 2.5.3. Cluster 3

Unlike the slow and continuous increase in protein content depicted in Cluster 2, proteins in Cluster 3 exhibited a fast accumulation at the early stage (day 1–3) of LL treatment, followed by a stabilization around the baseline level in the later stage of the treatment (Figure 3). This trend suggested that these proteins could be involved in the first cellular responses to LL treatment and TM biogenesis.

One significant functional class of proteins are phycobilisome components (Supplementary Table S7), which were tightly associated with TM even after several washes using low-salt buffers. They were accumulated faster than other photosynthetic components within Cluster 2. Phycobilisomes serve as the light-harvesting antenna of photosystems in cyanobacteria [82,83,84], and fast response to the change from HL to LL is essential to guarantee sufficient light absorption while light availability is reduced.

Proteins involved in amino acid biosynthesis, chlorophyll biosynthesis, nucleotide metabolism, and hydrolysis were also found in Cluster 3 (Supplementary Table S7). These processes and pathways function in providing basic building blocks for the synthesis of proteins and nucleic acids, which would be highly demanded by the increasing synthesis of photosynthetic components shown in Cluster 2. Furthermore, the increase in hydrolase activity could release energy stored in ATP to power the biosynthesis processes, suggesting active energy-consumption events happening during TM formation. Cluster 3 also comprises the components involved in translation, such as structural constituents of ribosomes (Supplementary Table S7). Among the 14 identified ribosomal proteins, 11 were grouped in Cluster 3. The results indicated that the first proteins quickly induced are those for the entire translation apparatus and those for central anabolic processes. Following a quick protein accumulation at this initial stage, a metabolic rewiring towards the photosynthetic lifestyle occurs.

#### 2.5.4. Cluster 4

Proteins in Cluster 4 exhibited a delayed increase at the later stage of LL adaptation, suggesting that the expression regulation of these proteins was a result of long-term response to LL. A significant group of proteins found in Cluster 4 are accessory components that are required for the assembly of the type IV pilus (T4P) appendages or protein secretion [85]. These include PilM (Q31KD6), PilO (Q31KD8), PilN (Q31KD7), and GspD (Q31KD9) (Supplementary Table S8). T4P is a cell-surface proteinaceous filament and carries out twitching motility to move away or toward a light source, contributing to fitness and survival. Genetic evidence suggested that T4P plays an important role in the transfer of newly synthesized chlorophylls in Syn6803 [86]. Recent bioinformatic analysis suggested the existence of a single complex that can affect protein secretion [87]. Based on their delayed increase during LL treatment, we propose that T4P accessory components including PilM, PilQ, PilN, and GspD are involved in protein secretion or chlorophyll transfer which were synthesized at the early stage of LL treatment in Cluster 3.

Secretion systems can transport proteins from the cytoplasm into other compartments of the cell, or the environment. Some secretion systems are highly conserved and widespread across all kingdoms, including the classical secretory (Sec) pathway and the twin-arginine translocation (TAT) pathway [88]. We found the SecY subunit (P0A4H0) [89] in Cluster 4, which is a component of the SecYEG complex facilitating the incorporation of chlorophylls into PSII [90].

FtsH metalloproteases are responsible for removing damaged PSII following photoinhibition [91]. Four FtsH isoform proteins (FtsH1, Q31RJ0; FtsH2, Q31PP7; FtsH3, Q31PJ1; FtsH4, Q31NM5) were characterized in our analysis (Supplementary Table S8), among which three (FtsH1, FtsH2, FtsH4) were in Cluster 4 that showed delayed accumulation under LL treatment. This result suggests that these three FtsH isoforms may not be involved in HL-induced D1 degradation, but could be largely responsible for quality control of PSII in GL. In contrast, FtsH3 may be involved in PSII turnover under HL, like its homolog in Syn6803 (Slr0228) [60,92].

#### 2.5.5. Cluster 5

Cluster 5 is the least populated group of all six, including 31 proteins that exhibit no clear trajectories in protein abundance changes; their protein expression oscillated around the baseline. Most of the proteins in Cluster 5 were involved in transcriptional regulation and metabolism (Supplementary Table S9).

#### 2.5.6. Cluster 6

The changes in protein content in Cluster 6 exhibited a fast decline in the first 1–3 days of LL treatment and then became consistent around the baseline level at the later stage. This trend could be an indication that proteins in Cluster 6 are positively responsive to HL acclimation but less responsive to long-term LL treatment. A significant class of proteins in Cluster 6 are membrane transporters, including Fe (III) ABC transporter FutC (Q31ND3) and high-affinity iron transporter Ftr1 (Q31KG8) (Supplementary Table S10), as discussed before. Respiratory terminal oxidases, such as cytochrome bd-type quinol oxidase Cyd (Q31MC3) and aa3-type Cyt c oxidase Cox (Q31JY7), were also found in Cluster 6. Their orthologs are responsible for adaptation to fluctuating light in Syn6803 [93]. Our results suggested functional conservation of the family proteins in cyanobacteria response to changing light intensity, in particular HL.

## 3. Conclusions

In summary, this study presents the global proteome analysis of TM protein complexes and structurally and functionally associated components from Syn7942 grown under HL, as well as the dynamic changes in protein abundance during light adaptation. A profound investigation of protein abundance profiles within TM provides valuable insights into the hierarchical integration of proteins during the TM biogenesis process and the in vivo regulation of bioenergetic and cellular reactions. Our results and analytical approaches may also facilitate the understanding of the response mechanisms of TM development and photosynthetic adaptations in plants and algae. Moreover, with the rapid development of artificial intelligence and machine learning, systematic bioinformatic analysis of the omics data will allow us to address many questions in cellular activities with unprecedented detail, such as the roles and dynamic abundance of hypothetical proteins, the significance of soluble proteins and lipids in TM and cellular membrane biogenesis, as well as the global adaptative responses of cells against various environmental stresses.

## 4. Materials and Methods

### 4.1. Strains and Culture Conditions

Cells of Syn7942 were grown at 30 °C in BG11 medium in culture flasks with constant shaking. The light conditions are 40 (GL), 300 (HL), and 20 (LL) μmol photons m^−2^ s^−1^, respectively. To determine proteome dynamics of membrane fractions [10], Syn7942 cells were cultured under continuous growth light (GL) for 5 days and treated with high light (HL) for 14 days until the stable minimal level of TM was reached. From that time point, TM regeneration was initiated by shifting cells to low light (LL). Cell growth under LL was monitored for 6 days until full recovery, and sample cells were harvested on each day (LL1–LL6) (Figure 1) [10]. All samples (GL, HL, LL1–LL6) had three biological replicates.

### 4.2. Membrane Preparation

The harvested Syn7942 cells were resuspended in isolation buffer (50 mM HEPES-NaOH, pH 7.5, 30 mM CaCl_2_, 800mM sorbitol, and 1 mM ε-amino-n-caproic acid), and disrupted by vortexing with glass beads (212–300 μm in diameter) at 4 °C. The glass beads and unbroken cells were removed by centrifugation at 3000× *g* for 5 min. From the total protein extracts, membranes and soluble proteins were fractioned by centrifugation at 17,000× *g* for 30 min and resuspended in storage buffer (50 mM Tricine-NaOH, pH 7.5, 600 mM sucrose, 30 mM CaCl_2_, and 1M glycinebetaine) [10].

### 4.3. Mass Spectrometry Measurement

Membrane fraction was reconstituted, reduced, alkylated, and digested as described before [10], with the exception that trypsin to protein ratio was 1:50. The following day, Rapigest was removed by the addition of 0.5% (*v*/*v*) TFA and incubation at 37 °C for 45 min. Digests were centrifuged at 17,200× *g* for 30 min and the clarified supernatants aspirated. Samples were stage-tipped on C18 filters to remove chlorophyll prior to LC-MS/MS analysis.

As reported previously [10], data-dependent LC-MS/MS analyses were conducted on a QExactive quadrupole-Orbitrap mass spectrometer coupled to a Dionex Ultimate 3000 RSLC nano-liquid chromatograph (Dionex/Thermo Fisher Scientific, Waltham, MA, USA). An equivalent of 100 ng peptides per sample was injected for mass spectrometry. The mass spectrometer was operated in DDA mode with survey scans from *m*/*z* 300−2000 acquired at a mass resolution of 70,000 (FWHM) at *m*/*z* 200. The ten most intense precursor ions with charge states of between 2+ and 5+ were selected for MS/MS with an isolation window of 2 *m*/*z* units.

Database search and protein identification: raw data files were searched against the UniProt proteomes database of Syn7942 (UniProt ID: UP000002717) using Proteome Discoverer software (ThermoFisher Scientific version 1.4.1.14) connected to an in-house Mascot server (Matrix Science, version 2.4.1, London, UK). A precursor ion tolerance of 10 ppm and a fragment ion tolerance of 0.01 Da were used with carbamidomethyl cysteine set as a fixed modification and oxidation of methionine as a variable modification.

Label-free quantification in Progenesis QI for MS: raw mass spectral data files were processed using Progenesis QI (v4.1; Nonlinear Dynamics, Edmonton, AB, Canada) to determine total protein abundances. All raw files were initially automatically aligned, according to the retention time, to produce an aggregate LC-MS map, from which peptide feature charge states +1 and >+7 were excluded. A precursor ion tolerance of 10 ppm and a fragment ion tolerance of 0.01 Da were used, with carbamidomethylation of cysteine set as a fixed modification and oxidation of methionine as a variable modification. Trypsin was the specified enzyme, and one missed cleavage was allowed. A peak list was exported to Mascot and searched against the UniProt proteomes database of Syn7942 (UniProt ID: UP000002717) using the Mascot search engine.

### 4.4. Data Analysis

All the analyses were performed using the R statistical computing environment (v.4.0) (R Core Team, Vienna, Austria, 2021). The data were log2 normalized using probabilistic quotient normalization (PQN) and batch corrected using Combat from the package sva [94]. All pairwise comparisons were performed using the “limma” method [95] with the Benjamini–Hochberg (BH) method for false discovery rate adjustment. Further statistical analysis included Spearman correlation tests for all proteins with respect to time (*p*-values adjusted by BH) and significant proteins used for functional enrichment.

Pathway and GO term enrichment analysis were performed using standard overrepresentation (ORA) enrichment analysis algorithm implementation in the R package “ClusterProfiler” [96]. Differentially abundant proteins were used as the target (foreground) set and all measured proteins as the background set with BH were used for false discovery rate adjustment.

The protein time-series clustering was carried out using the “dtwclust” method [97]. This method aims to cluster time-series data allowing for shifts along the time axis which results in clustering by the shape of the trend rather than just the individual numeric values at each time point. Prior to clustering, the initial time point data were subtracted from the rest of the time points and the results were converted to z-scores by subtracting the mean and dividing by the standard deviation of the protein at the corresponding time point. Clustering the data in z-score form resulted in groups of protein trends without taking into account absolute abundances and, instead, focusing on the overall shape of the response. This allowed an unbiased exploratory analysis of common protein trends. For protein function analysis, GO descriptions were used to acquire available annotations of the Syn7942 thylakoid proteome. Each cluster was functionally assigned manually based on all available annotations.

Protein structural characteristic predictions were made using DeepTMHMM v1 [24] and signal peptide presence predictions were made using SignalP v6 [25].

## Figures and Tables

**Table 1:** Photosynthetic complex proteins are significantly regulated under HL compared with GL. Up-regulated protein subunits are highlighted in yellow.

UniProt	Description	Fold Change	UniProt	Name	Fold Change
**PSII core**			**PSI core**		
Q31RR2	PsbH	0.092	Q31NT9	PsaF	0.130
P11004	PsbC (CP47)	0.210	Q31QV2	PsaC	0.135
P11472	PsbO	0.238	P95822	PsaL	0.155
P31094	PsbB (CP43)	0.241	Q31NU0	PsaJ	0.161
Q8KPP1	PsbL	0.250	Q5MZZ8	PsaM	0.164
P04996	PsbA1 (D1)	0.258	Q31PI7	PsaD	0.176
P11005	PsbD (D2)	0.267	Q31LJ1	PsaB	0.195
Q8KPP3	PsbE (Cyt *b*_559_)	0.284	Q31LJ0	PsaA	0.208
Q31LS7	PsbY	0.342			
P04997	PsbA2	2.255			
**PSII assembly factors**			**PSI assembly factors**		
Q31QR0	Psb34	0.300	Q31Q43	VIPP1	0.455
Q31RE4	Psb27	0.439	Q31NR5	Ycf37	0.631
Q31ML0	Psb28	0.693			
Q31KQ9	CtpA	0.530			
**Cyt *b*_6_*f***			**NDH-1**		
Q31NV8	PetA	0.236	Q31QG9	NdhP	0.365
Q54710	PetD	0.393	Q8VPV4	NdhF4	0.384
Q54711	PetB	0.400	Q8VPU9	NdhD4	0.466
Q31NV7	PetC	0.434	Q31NJ3	NdhE	1.974
			Q31LY1	NdhV	1.978
			Q8VPV6	CupS	2.663
			Q8VPV9	NdhF3	3.055
			Q8VPV7	CupA	5.060
			Q31LE7	NdhD3	12.812
**PBS**					
Q31PD9	CpcL	0.166			
Q31PE0	CpcH	0.259			
P06539	CpcB1	0.268			
Q31PD8	CpcD	0.269			
Q31RF9	ApcE	0.295			
Q31RG0	ApcA	0.305			
O50209	ApcF	0.328			
Q31RG1	ApcB	0.333			
Q31LK9	CpcG	0.342			
Q31RP7	ApcD	0.416			
Q31Q37	CpcT	0.621			

## Data Availability

Data are available within the article and Supplementary Materials. The proteome data of thylakoid membranes isolated from cells grown under different light conditions are available at the ProteomeXchange Consortium via PRIDE partner repository with the project accession PXD019731 (https://www.ebi.ac.uk/pride/archive/projects/PXD019731 (accessed on 17 November 2020). Other data will be available from the corresponding authors upon reasonable request.

## Supplementary Materials

The following supporting information can be downloaded at: https://www.mdpi.com/article/10.3390/plants12233967/s1, Figure S1: Boxplots of samples before and after data normalization via Probabilistic Quotient Normalization. Figure S2: Principal Component Analysis score plot of samples before and after data normalization. Figure S3: GO and KEGG pathway overrepresentation enrichment analysis results. Figure S4: ABC transporters identified in our analysis. Figure S5: Changes in protein abundance of cytochrome oxidases during TM biogenesis. Table S1: Total Proteins detected and quantified with multiple peptides identified. Table S2: Protein transmembrane structural predictions. Table S3: Signal peptide predictions. Table S4: Differentially regulated proteins between HL and GL. Table S5: Cluster 1 proteins. Table S6: Cluster 2 proteins. Table S7: Cluster 3 proteins. Table S8: Cluster 4 proteins. Table S9: Cluster 5 proteins. Table S10: Cluster 6 proteins.
