# Supplementary material for: Dynamic Changes in the Thylakoid Proteome of Cyanobacteria during Light-Regulated Thylakoid Membrane Development
Source: Plants (Basel). 2023 Nov 25;12(23):3967. doi: 10.3390/plants12233967 (PMC10708155; doi:10.3390/plants12233967)
Supplement: Supplementary file 1 [file plants-12-03967-s001.zip › plants-2714760-supplementary/plants-2714760-supplementary Figures.pdf]

Supplementary Information

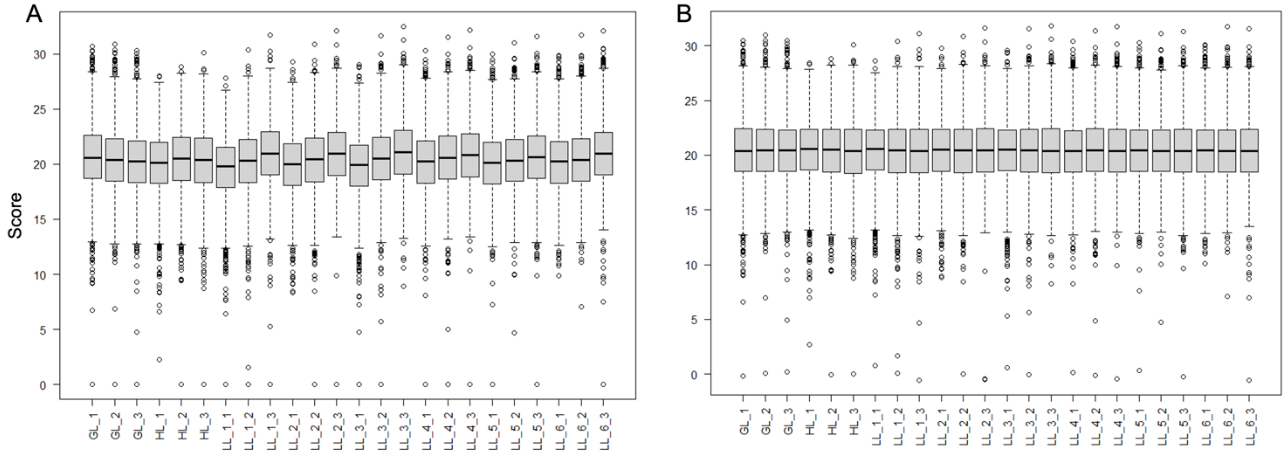

Supplementary Figure S1. Boxplots of samples before (A) and after (B) data normalization via Probabilistic Quotient Normalization (PQN). While in (A) we can see sample variation with a slight trend for replicate one to show lower abundance than replicate two and three, after normalization (B) we have minimized unwanted variance and all samples are more comparable in their distribution.

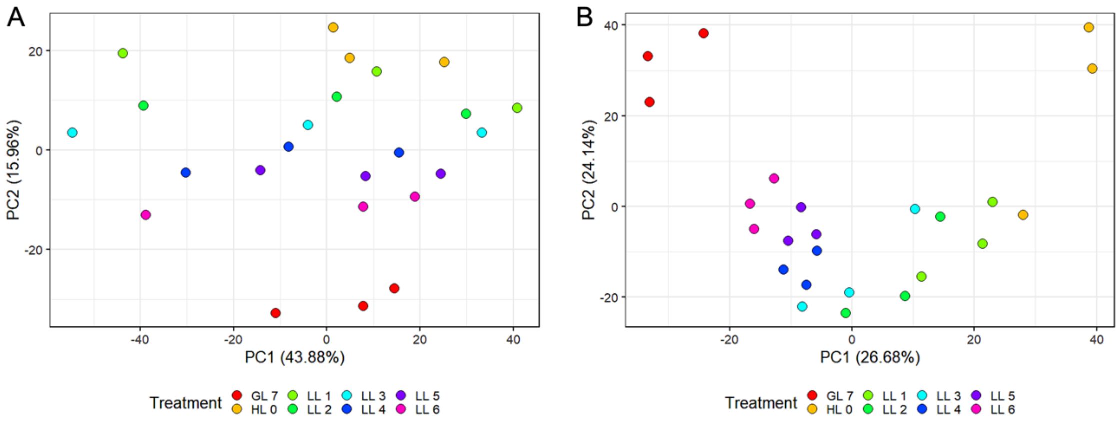

Supplementary Figure S2. Principal Component Analysis score plot of samples before (A) and after (B) data normalization. Each point represents a sample, colored based on the experimental treatment (High Light, HL; Growth Light, GL; Low Light, LL; time point, 0-7). It can be appreciated that in (A) most of the variance accounted for by PC1 is related to technical variation. After normalization PQN, this improves but technical variance still accounts for the most variance in PC1. After batch effect correction (B), we can see that PC1 mainly accounts for the treatment/time outcome variable and technical variation has been reduced, with the triplicates for each condition clustering together.

- Positive correlation

Term	Info	p.val adj	Description
GO:0015979	GO terms	5.4×10^{-5}	Photosynthesis
GO:0042651	GO terms	1.0×10^{-4}	Thylakoid membrane
syf00195	KEGG	7.3×10^{-13}	Photosynthesis
syf00196	KEGG	4.27×10^{-8}	Photosynthesis-antenna proteins

- Negative correlation

Term	Info	p.val adj	Description
syf0210	KEGG	0.028	ABC transporters

Supplementary Figure S 3. GO and KEGG pathway overrepresentation (ORA) enrichment analysis results. ORA was undertaken with the list of proteins that shown a positive correlation over time and negative correlation over time. P.val adj, p-value adjustment.

<table>
<tr><th>Proteins</th><th>GO_desc</th><th>logFC</th></tr>
<tr><td>O05347</td><td>membrane | ATP binding | ATPase activity | nitrate transmembrane transporter activity</td><td>1.0228182</td></tr>
<tr><td>P38043</td><td>plasma membrane | ion transport | nitrate assimilation</td><td>-1.5042405</td></tr>
<tr><td>Q31KE6</td><td>integral component of membrane | plasma membrane | inorganic phosphate transmembrane transporter activity | phosphate ion transport</td><td>1.3349057</td></tr>
<tr><td>Q31KE7</td><td>integral component of plasma membrane | inorganic phosphate transmembrane transporter activity | phosphate ion transmembrane transport</td><td>0.6947131</td></tr>
<tr><td>Q31KE8</td><td>plasma membrane | ATP binding | ATPase activity | ATPase-coupled phosphate ion transmembrane transporter activity | inorganic phosphate transmembrane transporter activity</td><td>0.8743749</td></tr>
<tr><td>Q31MQ0</td><td>ATP binding | ATPase activity</td><td>0.4741266</td></tr>
<tr><td>Q31ND2</td><td>integral component of membrane | plasma membrane | transmembrane transport</td><td>1.6290874</td></tr>
<tr><td>Q31ND3</td><td>ATP-binding cassette (ABC) transporter complex | ATP binding | ATPase activity | transmembrane transporter activity</td><td>1.7557573</td></tr>
<tr><td>Q31PM9</td><td>ATP binding | ATPase activity</td><td>0.6200510</td></tr>
<tr><td>Q31PP2</td><td>ATP binding | ATPase activity</td><td>-0.6545657</td></tr>
<tr><td>Q31Q22</td><td>ATP binding | ATPase activity</td><td>-0.8768304</td></tr>
<tr><td>Q31Q55</td><td>integral component of membrane | ATP binding | ATPase activity | ATPase-coupled transmembrane transporter activity</td><td>1.0001084</td></tr>
<tr><td>Q31QJ7</td><td>integral component of membrane | ATP binding | ATPase activity | ATPase-coupled transmembrane transporter activity</td><td>1.1407667</td></tr>
<tr><td>Q31R16</td><td>integral component of membrane | plasma membrane | carbohydrate transport | transmembrane transport</td><td>-0.8114216</td></tr>
<tr><td>Q31RD6</td><td>integral component of membrane</td><td>0.6186039</td></tr>
<tr><td>Q31RD7</td><td>ATP binding | ATPase activity</td><td>-0.5905658</td></tr>
<tr><td>Q31RP0</td><td>ATP-binding cassette (ABC) transporter complex | transmembrane transporter activity | amino acid transport</td><td>1.2434017</td></tr>
<tr><td>Q7BW13</td><td>integral component of membrane | plasma membrane | transmembrane transport</td><td>1.4901848</td></tr>
<tr><td>Q8GIU0</td><td>ATP binding | ATPase activity</td><td>-0.6728100</td></tr>
</table>

Supplementary Figure S4. ABC transporters identified in our analysis.

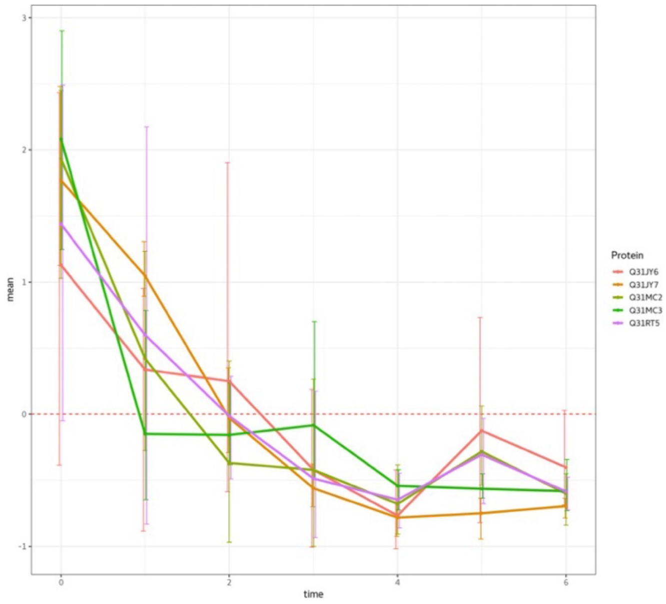

Q31JY6	Cytochrome c oxidase
Q31JY7	Cytochrome c oxidase subunit 2
Q31MC2	Cytochrome oxidase d subunit I
Q31MC3	Cytochrome d ubiquinol oxidase, subunit II
Q31RT5	Cbb3-type cytochrome oxidase subunit 1-like

Supplementary Figure S5. Changes in protein abundance of cytochrome oxidases during TM biogenesis. On the left shown mean Z-score abundance of the five Cyt oxidases over time with vertical bars representing standard deviation. On the right table showing protein identities with respect to accession numbers.
